# Diminishing Immune Responses against Variants of Concern in Dialysis Patients 4 Months after SARS-CoV-2 mRNA Vaccination

**DOI:** 10.3201/eid2804.211907

**Published:** 2022-04

**Authors:** Alex Dulovic, Monika Strengert, Gema Morillas Ramos, Matthias Becker, Johanna Griesbaum, Daniel Junker, Karsten Lürken, Andrea Beigel, Eike Wrenger, Gerhard Lonnemann, Anne Cossmann, Metodi V. Stankov, Alexandra Dopfer-Jablonka, Philipp D. Kaiser, Bjoern Traenkle, Ulrich Rothbauer, Gérard Krause, Nicole Schneiderhan-Marra, Georg M.N. Behrens

**Affiliations:** University of Tübingen Natural and Medical Sciences Institute, Reutlingen, Germany (A. Dulovic, M. Becker, J. Griesbaum, D. Junker, P.D. Kaiser, B. Traenkle, U. Rothbauer, N. Schneiderhan-Marra);; Helmholtz Centre for Infection Research, Braunschweig, Germany (M. Strengert, G. Krause);; TWINCORE GmbH Centre for Experimental and Clinical Infection Research, Hannover, Germany (M. Strengert, G. Krause);; Hannover Medical School, Hannover (G. Morillas Ramos, A. Cossmann, M.V. Stankov, A. Dopfer-Jablonka, G.M.N. Behrens);; Dialysis Centre Eickenhof, Langenhagen, Germany (K. Lürken, A. Beigel, E. Wrenger, G. Lonnemann);; German Centre for Infection Research, Hannover–Braunschweig, Germany (A. Dopfer-Jablonka, G. Krause, G.M.N. Behrens);; University of Tübingen Pharmaceutical Biotechnology, Tübingen (U. Rothbauer);; Centre for Individualized Infection Medicine, Hannover (G.M.N. Behrens)

**Keywords:** COVID-19, coronavirus disease, SARS-CoV-2, severe acute respiratory syndrome coronavirus 2, viruses, respiratory infections, zoonoses, vaccine-preventable diseases, dialysis, mRNA vaccination, variants of concern, booster dose, immunocompromised, longevity of immune response

## Abstract

Patients undergoing chronic hemodialysis were among the first to receive severe acute respiratory syndrome coronavirus 2 (SARS-CoV-2) vaccinations because of their increased risk for severe coronavirus disease and high case-fatality rates. By using a previously reported cohort from Germany of at-risk hemodialysis patients and healthy donors, where antibody responses were examined 3 weeks after the second vaccination, we assessed systemic cellular and humoral immune responses in serum and saliva 4 months after vaccination with the Pfizer-BioNTech BNT162b2 vaccine using an interferon-γ release assay and multiplex-based IgG measurements. We further compared neutralization capacity of vaccination-induced IgG against 4 SARS-CoV-2 variants of concern (Alpha, Beta, Gamma, and Delta) by angiotensin-converting enzyme 2 receptor-binding domain competition assay. Sixteen weeks after second vaccination, compared with 3 weeks after, cellular and humoral responses against the original SARS-CoV-2 isolate and variants of concern were substantially reduced. Some dialysis patients even had no detectable B- or T-cell responses.

Persistence of vaccination-induced cellular and humoral immune responses is crucial to prevent severe acute respiratory syndrome coronavirus 2 (SARS-CoV-2) infection or at least provide protection against severe coronavirus disease (COVID-19) that requires hospitalization. As in many other countries, the SARS-CoV-2 vaccination strategy in Germany was based on prioritization by occupation, underlying medical conditions, or advanced age (*1*). Although those priority groups have been vaccinated, a debate has emerged as to whether a third booster dose may be necessary to maintain or raise levels of protection within some of these groups. Decisions on whether to recommend a third dose needed to be made within a short timeframe, because SARS-CoV-2 infection case numbers were expected to increase again in the upcoming cold season, as previously observed in late 2020 (*2*). To date, however, data are lacking regarding the longevity of vaccination responses, and most published studies only provide follow-up data until 3 months after the second dose (*3*). Only 2 studies report data on extended time frames of 6 months after a completed 2-dose scheme (*4*,*5*), and, to our knowledge, no studies have considered follow-ups in patients receiving chronic hemodialysis. Data on the actual effect of a third dose are equally scarce and, so far, limited to organ transplant recipients, where a third dose substantially increased antibody responses (*6*). In addition, protection offered by first-generation vaccines is reduced for SARS-CoV-2 variants of concern (VOCs) (*7*), which now account for most infections worldwide (*8*), making the decision of whether a third dose is advisable even more critical for those with underlying conditions, immunodeficiencies, or an increased exposure risk (e.g., healthcare workers).

One particular risk group for SARS-CoV-2 infection and severe COVID-19 disease is hemodialysis patients; currently, ≈80,000 persons requiring regular renal replacement therapy in Germany (*9*). Their various underlying medical conditions and dialysis therapy often lead to a state of generalized immunosuppression (*10*). At the same time, these patients bear a continuous exposure risk because of the regular need for in-center hemodialysis therapy, which prevents them from self-isolating or reducing contacts to avoid infection. We and others have identified impaired cellular and humoral responses towards several viral vaccinations (e.g., SARS-CoV-2, influenza A, or hepatitis B) (*10*–*13*); however, there is a lack of longitudinal vaccination response studies against SARS-CoV-2 within this population. To guide future vaccination strategies as to whether additional booster vaccinations for at-risk groups to prevent severe COVID-19 are required, we provide follow-up data for a previously reported cohort of 76 persons receiving hemodialysis and 23 healthcare workers with no underlying conditions (*13*) for systemic and mucosal B- and T-cell responses 16 weeks after full BNT162b2 vaccination and the neutralizing potency of vaccination-induced antibodies. Because of the emergence of VOCs, and because all currently licensed vaccines are formulated against the original wild-type isolate (B.1), we also examined antibody binding and neutralization toward the Alpha (B.1.1.7), Beta (B.1.351), Gamma (P.3) and Delta (B.1.617.2) VOCs.

## Methods

### Study Design and Sample Collection

We collected blood samples by using vascular access before the start of dialysis or by venipuncture for the control population 16 weeks after the standard 2-dose vaccination with a 21-day interval of BNT162b2 (Pfizer-BioNTech, https://www.pfizer.com) was completed (T2). An analysis of samples from this population that were collected 3 weeks after the second dose of BNT162b2 (T1) has been published previously (*13*). A total of 76 patients on maintenance hemodialysis and 23 healthcare workers from the same dialysis center participated in the longitudinal follow-up (*13*). Demographic characteristics (e.g., age and sex), body mass index, time on dialysis, use of immunosuppressive medications, and anti-S1 domain IgG levels at T1 of persons who did not provide a sample at T2 were not substantially different compared with persons included in this analysis (Table; Appendix Table 1, 2). We obtained plasma by using an S-Monovette lithium heparin blood collection kit (Sarstedt, https://www.sarstedt.com). We used whole-blood samples immediately for an interferon-γ (IFN-γ) release assay (IGRA). To inactivate potential pathogens, we treated collected saliva samples with Tri (n-butyl) phosphate for a final concentration of 0.3% and Triton X-100 for a final concentration of 1%.

**Table:** Characteristics of participants in a study of immune response against variants of concern in dialysis patients 4 months after SARS-CoV-2 mRNA vaccination*

Characteristic	Nondialysis control group	Hemodialysis group	p value for difference between groups
No. (%) patients	23 (100)	76 (100)	NA
Median age, y (IQR)	55 (14)	70.5 (18.25)	2.78 × 10^−9^
Sex			1.01 × 10^−2^†
M	6 (26.09)	43 (56.58)	
F	17 (73.91)	33 (43.42)	
Median days since start of hemodialysis (IQR)	NA	1,337 (1,686.5)	NA
Using immunosuppressive medication	0	10 (13.16)	6.77 × 10^−2^
Underlying condition			
Obesity, BMI >30‡	4 (17.39)	16 (21.05)	8.68 × 10^−1^
Diabetes mellitus	0	19 (25)	7.30 × 10^−3^
Cardiovascular disease	0	35 (46.05)	2.93 × 10^−5^

*Values are no. (%) except as indicated. Percentages are for total group. BMI, body mass index; IQR, interquartile range; NA, not applicable; SARS-CoV-2, severe acute respiratory syndrome coronavirus 2. †p value reflects difference in male-to-female ratios between the two groups, not differences explicitly for either male or female persons. ‡BMI for 1 person was not known.

### Ethics Considerations

The study was approved by the Internal Review Board of Hannover Medical School (approval number 8973_BO-K_2020). We obtained written informed consent from all participants before the start of the study.

### Bead Coupling

We coupled antigens to spectrally distinct MagPlex beads (Luminex, https://www.luminexcorp.com) by using EDC/s-NHS coupling for all standard (MULTICOV-AB) antigens (*14*). We coupled receptor-binding domains (RBDs) from VOCs by using Anteo coupling (AnteoTech, https://www.anteotech.com) according to the manufacturer’s instructions (*15*).

### MULTICOV-AB

We analyzed IgG and IgA binding and levels by using MULTICOV-AB, a multiplex coronavirus immunoassay, as previously described (*14*). For our study, we used a panel of recombinant proteins as antigens (Appendix Table 3). In brief, we immobilized antigens on spectrally distinct populations of MagPlex beads either by EDC/s-NHS coupling (*14*) or by Anteo coupling according to the manufacturer instructions (*15*). We then incubated the combined MagPlex beads with samples. After conducting a wash step to remove unbound antibodies, we detected IgG or IgA with either R-phycoerythrin labeled goat anti-human IgG (Jackson ImmunoResearch, https://www.jacksonimmuno.com) or IgA (Jackson ImmunoResearch) as secondary antibodies. After conducting another wash step and bead resuspension, we measured samples once on a FLEXMAP 3D instrument (Luminex) by using the following settings: timeout, 80 s; gate, 7,500–15,000; reporter gain, standard photomultiplier tube; 40 events. Raw median fluorescence intensity (MFI) values or normalized values (MFI/MFI of quality control [QC] samples) (*15*) are reported. Three QC samples were measured per individual plate to monitor MULTICOV-AB performance. We measured all samples once.

### Angiotensin-Converting Enzyme 2 Receptor Binding Domain Competition Assay

We carried out an angiotensin-converting enzyme 2 receptor-binding domain (ACE2-RBD) competition assay as previously described (*15*; D. Junker et al., unpub. data, https://doi.org/10.1101/2021.08.20.21262328) to determine IgG neutralization capacity against SARS-CoV-2 wild-type and the VOCs. For this assay, we combined biotinylated ACE2 with individual samples (and as a control, ACE2 alone) and incubated with the previously described MULTICOV-AB bead mix. Before and after ACE2 detection with Streptavidin-PE (Moss, Fisher Scientific, https://www.fishersci.com), we conducted washes. We measured samples once on a FLEXMAP 3D instrument with the same settings as MULTICOV-AB and analyzed them by using normalization of MFI values against the control. We considered samples with a neutralization ratio <0.2 as nonneutralizing. This cutoff is based on comparison to a classic virus neutralization test (D. Junker et al., unpub. data).

### Euroimmun ELISA QuantiVac

As a control for the MULTICOV-AB results, we also analyzed plasma samples by using the Anti-SARS-CoV-2 QuantiVac ELISA IgG (Euroimmun, https://www.euroimmun.com). Samples were measured as previously described (*13*). We measured all samples once.

### IGRA

We analyzed SARS-CoV-2–specific T-cell responses from whole blood by measuring IFN-γ production after stimulation with a peptide pool from the SARS-CoV-2 spike S1 with the SARS-CoV-2 Interferon Gamma Release Assay (Euroimmun) and the IFN-γ ELISA (Euroimmun), as previously described (*13*). We subtracted background signals from negative controls and calculated final results in milli-IU (mIU) per milliliter by using standard curves. Results from positive and negative controls were not statistically significantly different between timepoints T1 and T2. We considered IFN-γ concentrations >200 mIU/mL as reactive. We defined this arbitrary cutoff by using average background IFN-γ activity without antigen-stimulation in all samples of T1 multiplied with 10 for the threshold for IGRA positive. Using this cutoff, we found negative IGRA results in all of the 15 control samples (prepandemic persons) (*16*). The upper limit of reactivity was 2,000 mIU/mL.

### Data Analysis and Statistics

We matched sample metadata and collecting results from different assay platforms in Microsoft Excel 2016 (https://www.microsoft.com). We used GraphPad Prism 8.4.3 (https://www.graphpad.com) for statistical analysis. We generated figures in RStudio 1.2.5001 running R 3.6.1 (https://www.rstudio.com). We used the beeswarm add-on package to visualize data as strip charts with overlaying boxplots and to create nonoverlaying datapoints and used the RcolorBrewer add-on to generate specific colors for plots. We then edited the figures by using Inkscape 0.92.4 (https://inkscape.org).

## Results

### Substantial Decrease in Antibody Titers from 3 Weeks to 4 Months Postvaccination

**Figure 1:**
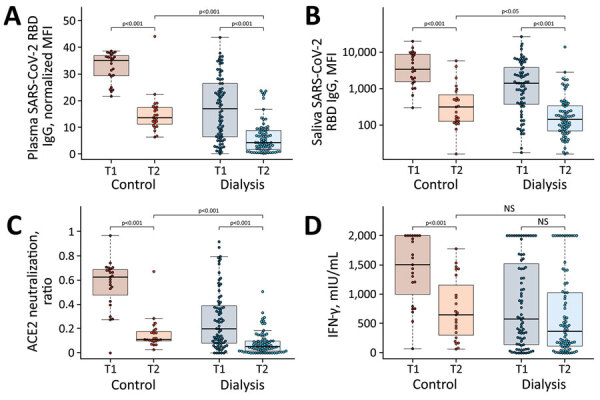
Significant decrease in humoral and cellular responses induced by Pfizer-BioNTech vaccine BNT162b2 (https://www.pfizer.com) against SARS-CoV-2 from 3 weeks to 16 weeks after second vaccination, observed in a study of immune response against variants of concern in dialysis patients 4 months after SARS-CoV-2 mRNA vaccination. A) IgG response in plasma; B) IgG response in saliva; C) neutralizing capacity toward SARS-CoV-2 wild type B.1; D) T-cell response measured by IFN-γ release assay. Blue circles indicate dialysis patients (n = 76) and red circles controls (n = 23). Samples were taken 3 weeks (T1) and 16 weeks (T2) after vaccination. Saliva (panel B) has reduced sample numbers in both groups because of issues in sample collection (T1 control, n = 22; T1 dialysis, n = 69; T2 control, n = 23; T2 dialysis. n = 71). T1 timepoint data has been published previously (*13*) and is reproduced here for clarity. Horizontal lines within boxes indicate medians; box tops and bottoms indicate the 25th and 75th percentiles; whiskers show the largest and smallest nonoutlier values. Outliers were determined by 1.5 times interquartile range. Statistical significance was calculated by Wilcoxon matched-pairs signed rank test when comparing T1 and T, and 2-sided Mann–Whitney–U test when comparing control and dialysis groups. ACE2, angiotensin-converting enzyme 2; IFN-γ, interferon γ; MFI; median fluorescence intensity; NS, not significant; RBD, receptor-binding domain; SARS-CoV-2, severe acute respiratory syndrome coronavirus 2; T1, timepoint 1; T2, timepoint 2.

Because antibody levels are considered a proxy for protection, we initially examined the seroreversion rate by using MULTICOV-AB (*14*), a previously published bead-based multiplex immunoassay that simultaneously analyses >20 different SARS-CoV-2 antigens, including the RBDs of VOCs and the endemic human coronaviruses. Similar to findings from our previous report (*13*), RBD IgG responses within the dialysis group (median normalized MFI 4.26 among 76 patients) toward SARS-CoV-2 wild-type RBD were significantly reduced compared with those for the control group (median normalized MFI 13.6 among 23 persons; p<0.001) (Figure 1, panel A) 16 weeks after complete vaccination (T2). Compared with titer levels at 3 weeks after the second dose (T1), at 16 weeks after (T2), antibody titers had significantly decreased, by 61% in the control group and 75% in the dialysis group (p<0.001) (Figure 1, panel A). RBD IgG levels measured by MULTICOV-AB were additionally verified with a commercial quantitative in vitro diagnostic antibody test (Spearman rank 0.956) (Appendix Figure 1). Although none of the samples of the control group were classified as seronegative (titer below the cutoff) (Appendix Figure 2), 19.7% (15/76) of dialysis samples were defined as such 16 weeks after the second dose (T2), which constitutes a substantial increase from 3 weeks after second vaccination (T1), at which point only 5.3% (4/76) of samples were seronegative. When examining plasma titers against nucleocapsid, we did not observe any dialysis patients, other than one who had a PCR-confirmed infection before the first dose, having a value above the cutoff that would indicate infection.

To evaluate whether this reduction in plasma RBD IgG was also present at the mucosal site, we profiled the local antibody response in saliva by using MULTICOV-AB. As observed in plasma, a significant reduction occurred in saliva RBD IgG titers in the dialysis (median 143 among 71 patients) compared with the control group (median 313.5 among 23 persons) (p = 0.02) (Figure 1, panel B). When comparing saliva RBD IgG levels at T1 to those at T2, we observed a statistically significant decline in both groups (p<0.001) (Figure 1, panel B), suggesting that the antibodies have potentially lost competence to prevent transmission if infected. When examining RBD IgA, we observed a significant difference in titers between persons in the control and dialysis groups (p = 0.003) (Appendix Figure 3, panel A); 47.8% of controls and 75% of dialysis patients were classified as seronegative. This more pronounced reduction in IgA versus IgG levels most likely represents the shorter IgA half-life. Saliva RBD IgA tended to be higher in the dialysis group, although not significantly (p = 0.051) (Appendix Figure 3, panel B).

### Decreased Neutralization Capacity as Time Postvaccination Increased

We next examined whether neutralization potential was also hindered because solid evidence exists on the protective role for neutralizing serum antibodies (*17*). By using an ACE2–RBD competition assay, which assesses neutralization potency toward SARS-CoV-2 wild-type and the circulating Alpha, Beta, Gamma, and Delta VOCs, we found that neutralization against wild-type SARS-CoV-2 RBD was significantly reduced in the dialysis group compared with the controls (p<0.001) (Figure 1, panel C) 16 weeks after complete vaccination. We found that 82.6% (19/23) of control samples and 89.5% (68/76) of dialysis patient samples were below the 0.2 threshold, which indicates the absence of neutralizing activity (Appendix Figure 2), a threshold is based on information provided for other available ACE2 competition assays (*18*). This difference represents a substantially significant reduction (p<0.001 for both groups) in neutralizing activity compared with 3 weeks after second vaccination, at which point only 4.3% (1/23) of the control samples and 50.0% (38/76) of the dialysis patient samples were below the threshold (Figure 1, panel C; Appendix Figure 2).

### Reduced T-cell Response after Vaccination in Dialysis Patients and Decrease Over Time

Because some persons might be able to control and clear SARS-CoV-2 infections with a strong T-cell response alone, we examined spike-specific SARS-CoV-2 T-cell responses by using a commercially available IGRA. Although absolute mean IFN-γ responses in the dialysis group compared with the control group tended to be lower (median 370 vs. 651 mIU/mL), this difference was not significant (p = 0.13) (Figure 1, panel D). In the control group, IFN-γ release after restimulation declined significantly from the first timepoint (median 1,505; p<0.001) (Figure 1, panel D), whereas for dialysis patients, this decline was not significant (median 580; p = 0.13) (Figure 1, panel D). This difference is probably attributable to most control samples being at the assay’s upper limit of detection at the first timepoint, when the dialysis samples already showed reduced IFN-γ release. Overall, the number of nonresponders was higher in the hemodialysis group (40.8% [31/76]) than the control group (21.7% [5/23]) (Appendix Figure 2). A lack of serologic response appears to be more driven by T-cell immunity than B-cell immunity; 2.6% (2/76) of the dialysis group having a T-cell response but no B-cell response, compared with 23.6% (18/76) who had a B-cell response but no T-cell response. In total, 17.1% (13/76) of the dialysis group were classified as complete nonresponders because of the absence of detectable SARS-CoV-2 wild-type B- and T-cell responses, compared with none in the control group.

### Significantly Reduced Antibody Binding and Neutralization Capacity against VOCs

**Figure 2:**
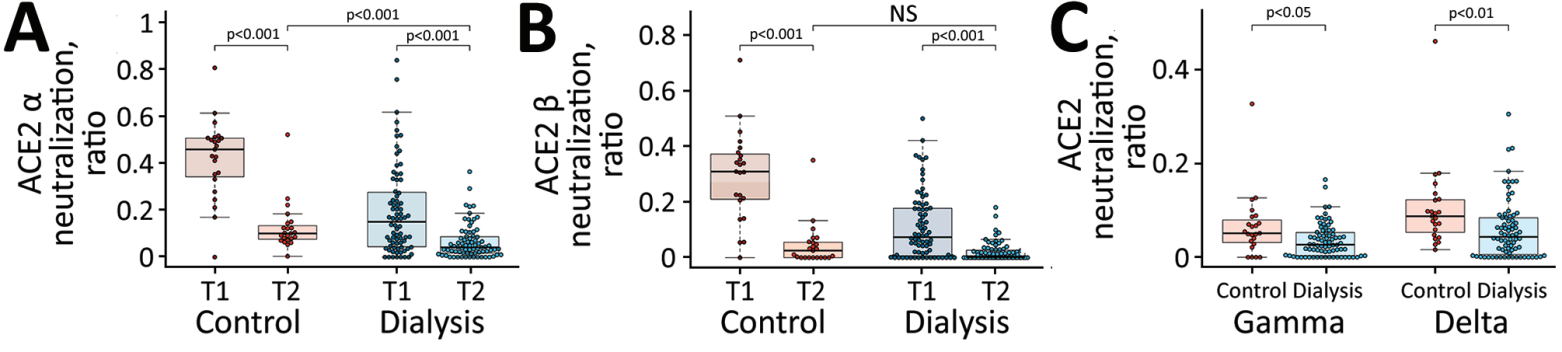
Reduced neutralizing capacity against SARS-CoV-2 variants of concern observed in a study of immune response against variants of concern in dialysis patients 4 months after SARS-CoV-2 mRNA vaccination. Neutralizing capacity of plasma IgG toward SARS-CoV-2 variants of concern Alpha (A), Beta (B), and Gamma and Delta (C) in the dialysis (blue circles, n = 76) and control (red circles, n = 23) groups 16 weeks after second vaccination with Pfizer-BioNTech vaccine BNT162b2 (https://www.pfizer.com). Neutralization capacity is displayed as ratio, where 1 indicates maximum neutralization and 0 no neutralization. Horizontal lines within boxes indicate medians; box tops and bottoms indicate the 25th and 75th percentiles; whiskers show the largest and smallest nonoutlier values. Outliers were determined by 1.5 times interquartile range. Statistical significance was calculated by 2-sided Mann–Whitney–U test. ACE2, angiotensin-converting enzyme 2; NS, not significant; SARS-CoV-2, severe acute respiratory syndrome coronavirus 2; T1, timepoint 1; T2, timepoint 2.

Having characterized response against wild-type SARS-CoV-2, we then assessed humoral response against the VOCs Alpha, Beta, Gamma, and Delta. As shown with classical cell-culture based virus neutralization assays (*7*), neutralization responses were also reduced for all VOCs compared with wild-type when we used the previously described ACE2-RBD competition assay. Compared levels at with the initial timepoint, neutralization decreased significantly for the Alpha and Beta VOCs (p<0.001 for both) (Figure 2, panel A, B). We were unable to determine these changes for Gamma and Delta because these variants were not measured in the initial analysis. In a comparison between the dialysis and the control cohort, dialysis patients had significantly reduced neutralization against Alpha (p<0.001) (Figure 2, panel A), Gamma (p = 0.014) (Figure 2, panel C), and Delta (p = 0.002) (Figure 2, panel C) but not for Beta (p = 0.08) (Figure 2, panel B). The number of nonresponders was variable between the different strains although consistently high; 87.0% of the control group and 93.4% of the dialysis group were considered nonresponders against Alpha, 95.7% of the control group and 100% of the dialysis group against Beta and Gamma, and 95.7% of the control group and 96.1% of dialysis group against Delta.

## Discussion

After our initial study (*13*), which focused on humoral and cellular responses 3 weeks after administering the second BNT162b2 vaccination, we provide longitudinal data for 4 months after the second dose. In comparison with other vaccine studies, which have mostly examined peak humoral response within 1 month or alternative prime-boost vaccination schedules with BNT162b2 (*12*), our data reveal a substantial decrease in the subsequent months in hemodialysis patients and healthy controls. Overall, the decline in neutralizing anti–spike RBD antibodies was comparable in both groups, and the difference between groups was mostly driven by differences in the magnitude of the initial humoral response. Although this decrease is expected and can be attributed to the memory phase, the extent of the reduction was unpredicted because it resulted in a substantial proportion of persons being classified as seronegative. The reduction of salivary antibodies is particularly important because their presence has been linked to reduced transmission potential (*15*). This pattern of reduced antibody binding with increasing time postvaccination was also reflected in diminishing neutralization potential.

Most persons tested were classified below our defined neutralization threshold for wild-type RBD with an almost complete nonresponder rate against Delta, which was the dominant strain in many parts of the world at the time of our analysis (*8*). Although this finding does not automatically translate to a failure of vaccine efficacy, given that any active challenge of the immune system should result in expansion of memory B- and T-cell populations along with increased (neutralizing) antibody titers, it does suggest nevertheless that active protection against infection may be reduced. Although a recent study by Pfizer (*4*) indicated that BNT162b2 vaccine efficacy did only slightly decrease 6 months postvaccination in the study cohort (from 95% to 91%) in fully immunocompetent persons, data from vaccinations in Israel identified a reduction in efficacy to 40% (*19*). In combination with our data, where 17.1% of the dialysis cohort were classified as having no evidence for vaccine-elicited T- and B-cell immunity after 4 months, the Pfizer study findings suggest that vaccine efficacy may be even further reduced within this patient group. For dialysis patients, this finding is particularly concerning because they often have underlying conditions that put them at additional risk for severe COVID-19 (*10*). The lack of a considerable SARS-CoV-2 specific T-cell response in dialysis patients may result from chronic inflammatory conditions, leading to T-cell exhaustion and suppression of IFN-γ levels (*20*). Differences in anti–SARS-CoV-2 T-cell kinetics between groups presumably reflect difference in the magnitude of T-cell responses after boost and during the contraction phase. To what extent T-cell immunity contributes to protection from COVID-19 and whether our IGRA results below a cutoff provide evidence for the lack of effective adaptive T-cell immunity, requires further investigation. However, we should state that although we see reductions in titer, neutralizing activity, and T-cell responses, we did not see any new infections by T2 within our cohort.

Our study is limited by the relatively small sample size of persons, who were not matched by age or sex. However, the sample number and compromised matching is consistent with similar studies on dialysis vaccine responses (*12*). Although studies have indicated that differences exist in protection and antibody responses (*21*) after different COVID-19 vaccination schedules, our study of Pfizer’s BNT162b2 represents a real-world situation for most dialysis patients. Because of reduced anti-spike responses 4 weeks postvaccination in patients with other chronic conditions (*6*), these groups should undergo careful monitoring to determine whether their responses also decrease substantially over time.

Taken together, our results strongly argue that all persons undergoing chronic hemodialysis should be preferably administered a third dose of the BNT162b2 vaccine. Recent studies on administering a third dose to dialysis patients and transplant recipients has identified strong increases in humoral responses after vaccination, and a reduced percentage of recipients are considered nonresponders (*22*–*25*). However, longitudinal follow-up studies will be needed in early 2022 to monitor the rate of antibody decay after administration of a third dose in these and other vulnerable groups.

Appendix Additional information about diminishing immune responses against variants of concern in dialysis patients 4 months after SARS-CoV-2 mRNA vaccination.
