# Supplementary material for: Diminishing Immune Responses against Variants of Concern in Dialysis Patients 4 Months after SARS-CoV-2 mRNA Vaccination
Source: Emerg Infect Dis. 2022 Apr;28(4):743–50. doi: 10.3201/eid2804.211907 (PMC8962909; doi:10.3201/eid2804.211907)
Supplement: Appendix — Additional information about diminishing immune responses against variants of concern in dialysis patients 4 months after SARS-CoV-2 mRNA vaccination. [file 21-1907-Techapp-s1.pdf]

Article DOI: https://doi.org/10.3201/eid2804.211907

Diminishing Immune Responses against Variants of Concern in Dialysis Patients 4 Months after SARS-CoV-2 mRNA Vaccination

Appendix

Appendix Table 1. Therapeutic indication for hemodialysis*

Diagnosis	No. patients (%) in hemodialysis group
Total	76 (100)
Autosomal dominant polycystic kidney disease	11 (14.47)
Chronic glomerulonephritis	6 (7.90)
Diabetic nephropathy	11 (14.47)
Focal segmental glomerulosclerosis	5 (6.59)
IgA nephropathy	8 (10.53)
Interstitial nephropathy	6 (7.90)
Nephrosclerosis	16 (21.05)
Acute toxic tubular epithelial damage syndrome	1 (1.32)
Primary amyloidosis	1 (1.32)
ANCA-associated vasculitis	1 (1.32)
Cardiorenal syndrome	2 (2.64)
Medullary cystic kidney disease	1 (1.32)
Membranous glomerulonephritis	1 (1.32)
Kidney dysplasia	1 (1.32)
Obstructive nephropathy	1 (1.32)
Reflux nephropathy	1 (1.32)
Septic organ failure	1 (1.32)
Cystic kidney disease	1 (1.32)
Cyclosporin intoxication	1 (1.32)

*ANCA, antineutrophilic cytoplasmic autoantibody.

Appendix Table 2. Medication of vaccination cohort

	No. (%)	
Medication	Nondialysis control group	Hemodialysis group
Total	23 (100)	76 (100)
Angiotensin-converting enzyme inhibitors	2 (8.70)	22 (28.95)
Statins	0 (0)	45 (59.21)
Angiotensin II Receptor Blockers	5 (21.74)	25 (32.89)
Vitamin D Supplements	12 (52.17)	75 (98.68)
Immunosuppressants (dosing range per day)*		
Prednisolone (2–7.5 mg)	0	5 (6.59)
Prednisolone (50 mg) day 6–14 post 2nd vaccination	0	1 (1.32)
Prednisolone (5 mg), Tacrolimus (0.5–2 mg)	0	2 (2.64)
Prednisolone (5 mg), Tacrolimus (12 mg), Mycophenolatmofetil (500 mg)	0	1 (1.32)
Hydrocortisone (20 mg)	0	1 (1.32)

*Therapeutic indication for immunosuppression were in four patients a kidney transplant (one had received an additional liver transplant), polymyositis, polyarthritis, vasculitis and chronic obstructive pulmonary disease.

Appendix Table 3. MULTICOV-AB antigen panel

Virus	Antigen	Manufacturer	Product number
SARS-CoV-2	Spike Trimer	NMI	–
SARS-CoV-2	RBD B.1 (wild-type)	NMI	–
SARS-CoV-2	Nucleocapsid	Aalto	6404–b
SARS-CoV-2	S1 domain	NMI	–
SARS-CoV-2	S2 domain	NMI	–
SARS-CoV-2	RBD B.1.1.7 (Alpha)	NMI	–
SARS-CoV-2	RBD B.1.351 (Beta)	NMI	–
SARS-CoV-2	RBD P.3 (Gamma)	NMI	–
SARS-CoV-2	RBD B.1.617.2 (Delta)	NMI	–
hCoV-OC43	S1 domain	NMI	–
hCoV-OC43	Nucleocapsid	NMI	–
hCoV-HKU1	S1 domain	NMI	–
hCoV-HKU1	Nucleocapsid	NMI	–
hCoV-NL63	S1 domain	NMI	–
hCoV-NL63	Nucleocapsid	NMI	–
hCoV-229E	S1 domain	NMI	–
hCoV-229E	Nucleocapsid	NMI	–

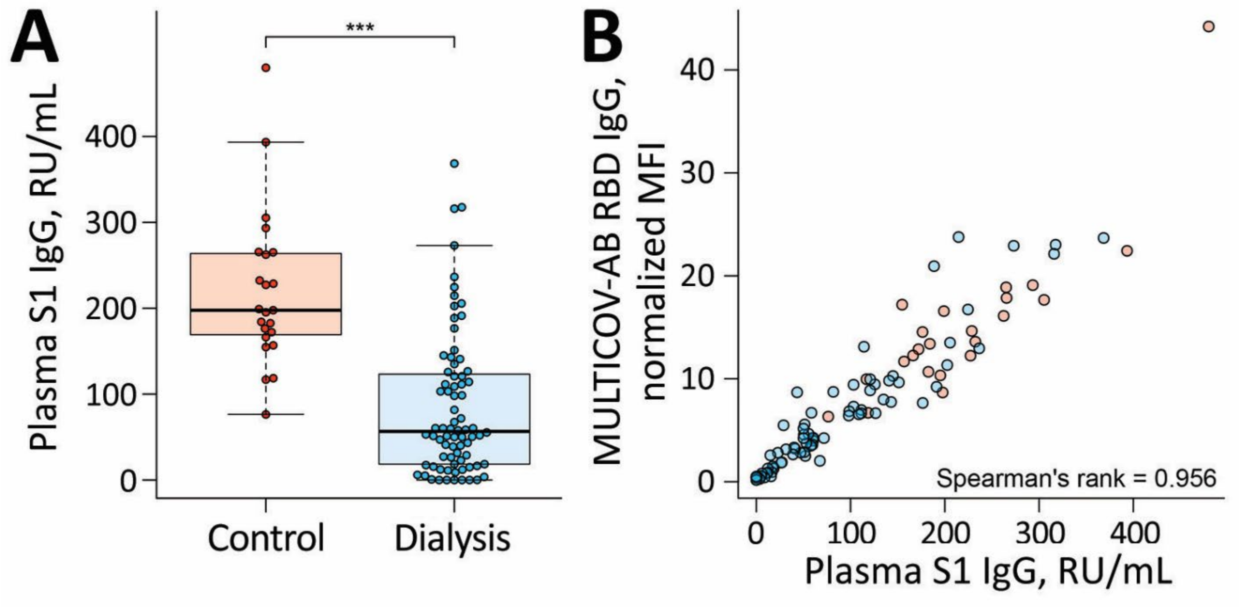

Appendix Figure 1. Quantitative plasma IgG titers 16 weeks after vaccination with Pfizer BNT162b2. A) Spike S1-specific plasma IgG (RU/mL) from control group (red, n = 23) and dialysis group (blue, n = 76) were analyzed 16 weeks post-second dose of Pfizer BNT162b2 using the QuantiVac-ELISA (Euroimmun). Samples above upper or below the ELISA's limits of detection are shown at the corresponding limit. Boxes represent the median, 25th and 75th percentiles, whiskers show the largest and smallest non-outlier values. Outliers were determined by 1.5 times IQR. Statistical significance was calculated by two-sided Mann-Whitney-U test. Significance was defined as ***<0.001. B) Correlation of MULTICOV-AB wild-type RBD B.1-IgG and QuantiVac Spike S1-IgG across the study population. Spearman's rank was used for correlation analysis.

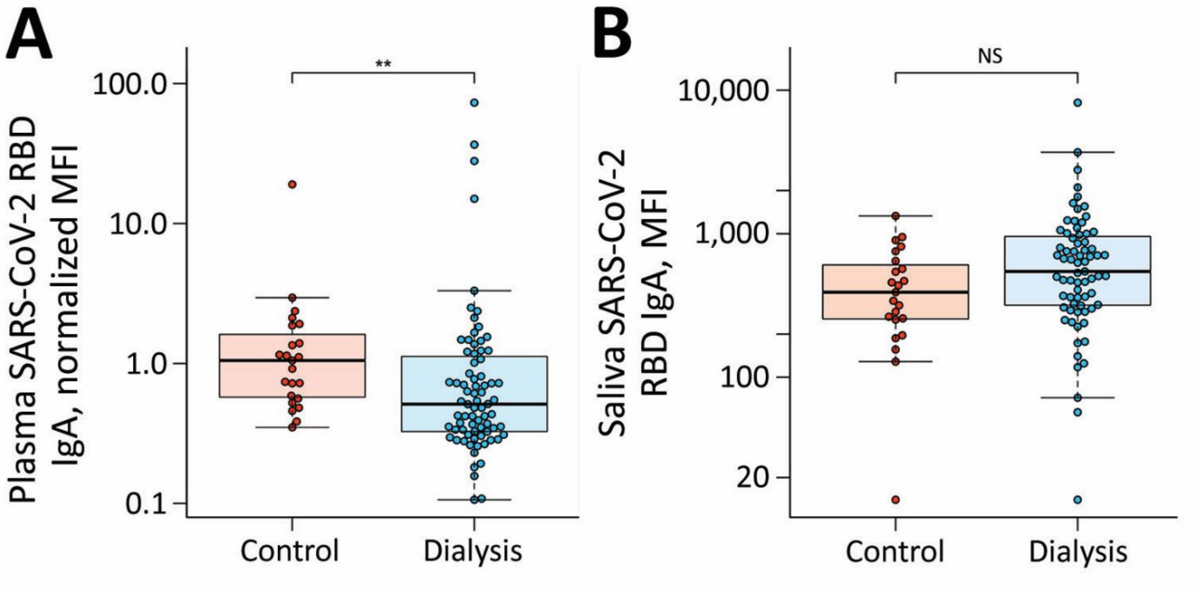
A
B
Plasma SARS-CoV-2 RBD IgA, normalized MFI
100.0
10.0
1.0
0.1
**
Control
Dialysis
Saliva SARS-CoV-2 RBD IgA, MFI
10,000
1,000
100
20
NS
Control
Dialysis

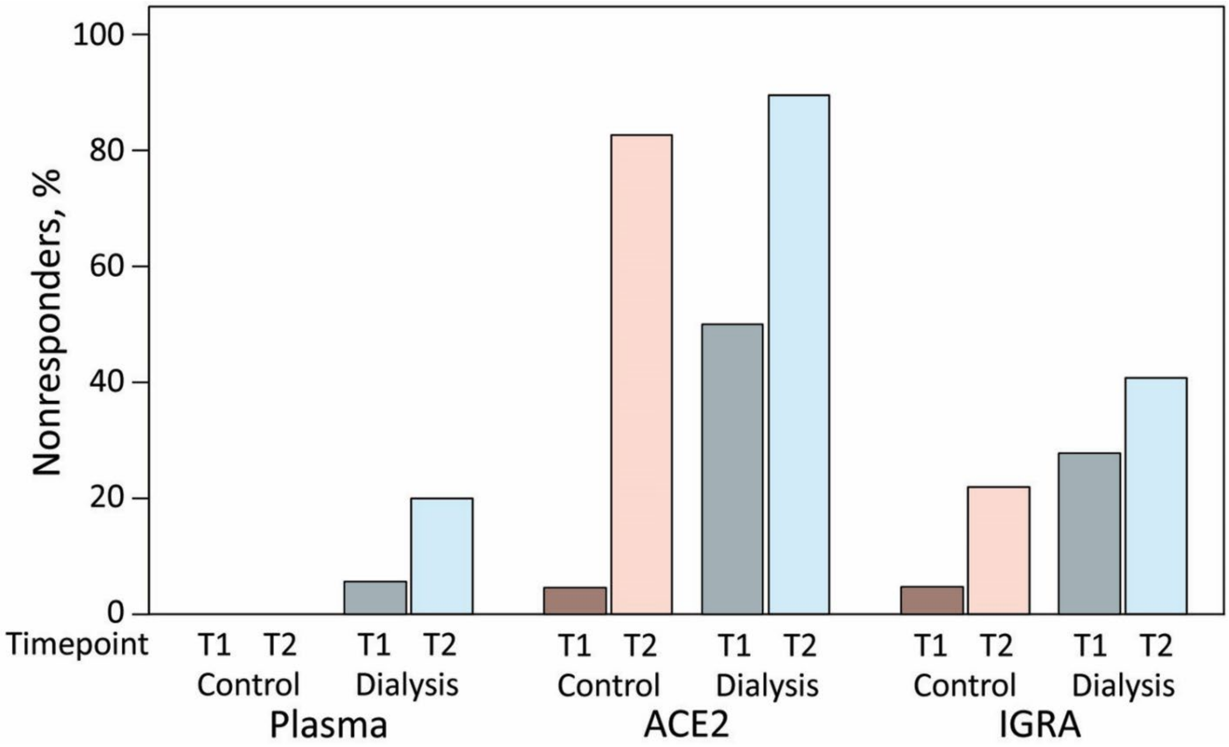

Reference

1. Strengert M, Becker M, Ramos GM, Dulovic A, Gruber J, Juengling J, et al. Cellular and humoral immunogenicity of a SARS-CoV-2 mRNA vaccine in patients on haemodialysis. EBioMedicine. 2021;70:103524. PubMed https://doi.org/10.1016/j.ebiom.2021.103524
